# The role of the mTOR pathway in diabetic retinopathy

**DOI:** 10.3389/fmed.2022.973856

**Published:** 2022-11-01

**Authors:** Fabio Casciano, Enrico Zauli, Erika Rimondi, Marco Mura, Maurizio Previati, Massimo Busin, Giorgio Zauli

**Affiliations:** ^1^Department of Translational Medicine and LTTA Centre, University of Ferrara, Ferrara, Italy; ^2^Interdepartmental Research Center for the Study of Multiple Sclerosis and Inflammatory and Degenerative Diseases of the Nervous System, University of Ferrara, Ferrara, Italy; ^3^Department of Translational Medicine, University of Ferrara, Ferrara, Italy; ^4^Research Department, King Khaled Eye Specialist Hospital, Riyadh, Saudi Arabia

**Keywords:** mammalian target of rapamycin (mTOR), diabetic retinopathy, hyperglycemia, reactive oxygen species (ROS), oxidative stress, inflammation, autophagy, retinae

## Abstract

The retina, the part of the eye, translates the light signal into an electric current that can be sent to the brain as visual information. To achieve this, the retina requires fine-tuned vascularization for its energy supply. Diabetic retinopathy (DR) causes alterations in the eye vascularization that reduce the oxygen supply with consequent retinal neurodegeneration. During DR, the mammalian target of rapamycin (mTOR) pathway seems to coordinate retinal neurodegeneration with multiple anabolic and catabolic processes, such as autophagy, oxidative stress, cell death, and the release of pro-inflammatory cytokines, which are closely related to chronic hyperglycemia. This review outlines the normal anatomy of the retina and how hyperglycemia can be involved in the neurodegeneration underlying this disease through over activation or inhibition of the mTOR pathway.

## Introduction

The retina is an anatomical site where microvascular architecture and neuronal organization are strictly related for proper visual function. Among ocular diseases, diabetic retinopathy (DR) is an increasingly prevalent degenerative disease and is the leading cause of blindness in developed countries. DR is one of the disorders related to diabetes mellitus (DM). DM is a chronic metabolic disease with multiple homeostatic alterations leading to the disruption of redox regulation, the activation of immune responses, and systemic inflammation (1). During DM, sustained hyperglycemia is a well-recognized cause of retinal microvascular/neuronal rewiring. As a consequence of hyperglycemia-induced energy imbalance, cells quickly alter their biochemical activity by enhancing the expression of advanced glycation end products (AGEs) and reactive oxygen species (ROS) (2). The kinase, mammalian target of rapamycin (mTOR), resides at the interface between hyperglycemia and biochemical modifications. mTOR is a sensor of nutrient availability and growth factors, and has been implicated in multiple diseases like cancer, diabetes, and aging (3). mTOR and its related pathways, namely, mTOR complexes (mTORCs), control tissue homeostasis to manage cell growth, proliferation, autophagy, and apoptotic events by virtue of its role. Indeed, changes in retinal morphology are driven by mTOR pathways, mainly by ROS production and dysregulated autophagic processes (4, 5).

This review addresses how hyperglycemia alters mTOR pathways during DR and provides useful tools for understanding normal retinal anatomy and the role of mTOR in tissue generation and in the pathophysiology of DR.

## Functional anatomy of the retina and blood–Retina barrier

The retina is the innermost of the three layers that constitute the wall of the eyeball. The retina internally covers the choroid and externally coats the vitreous body. Based on histological sections, the retina contains 10 different conventionally recognized morphological layers, composed of a complex array of neurons, glial cells, and blood vessels.

The first and outermost layer consists of the retinal pigment epithelium (RPE), which separates the retina from the innermost layer of the choroid, Bruch's membrane. More internally, three different types of neural cells are connected in series. Photoreceptors, rods, and cones convert the absorbed light into a neural signal, accounting for roughly 110–130 million cells in the entire retina. Photoreceptors, which account for roughly 30 million cells, are radially connected to bipolar cells, and transfer the neural signal to the innermost layer of ganglion cells whose axon forms the optic nerve, and are thought to number between 0.6 and 1.2 million. Physiological convergence of the signal from the more abundant photoreceptors to fewer ganglion cells is modulated by two different types of cells embedded in the retinal wall, horizontal cells that mediate the interaction between photoreceptors and bipolar cells, and amacrine cells interposed between bipolar cells and ganglion cells (6).

In addition to these five different types of neurons, the retina contains a large number of glial cells, namely, Müller cells, astrocytes, and microglia (7). Müller cells are the most representative and abundant among retinal glial cells. They constitute a larger part of the volume of the retina and fill the remaining space among neurons. Müller cells run radially from the inner to the outer limiting membrane, traversing all layers up to the outer nuclear layer and isolating neurons from each other except at their synaptic contacts. In correspondence with the internal limiting membrane, Müller cells contribute to the formation of this membrane with a footplate formed by their basal expansions. Astrocytes are much less abundant than Müller cells and are instead confined to ganglion cell and nerve fiber layers, while microglial cells are distributed throughout the whole retinal thickness but are particularly found near the vessels. It has also been demonstrated that glial cells are involved in retinal inflammation. Indeed, Müller cells have different receptors for cytokines and release cytokines to regulate inflammation (8). In physiological conditions, microglial cells maintain the homeostasis of the retina, undertake phagocytosis, clear debris, and control inflammation. Prolonged stress conditions, such as hyperglycemia associated with DR, can increase the number of microglial cells and release stress proteins and cytokines (9).

**Figure 1:**
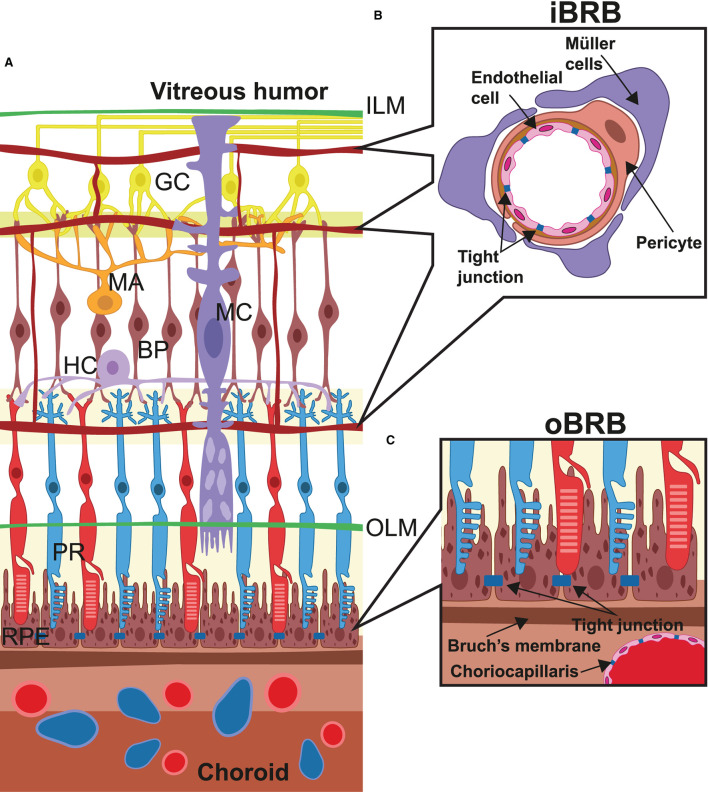
Retinal microanatomy and blood–retina barriers. **(A)** This image depicts the retinal structure situated in between the internal limiting membrane (ILM) and the choroidal layer (choroid), which externally lines the retinal pigment epithelium (RPE) cells. Over the RPE layer, neurons cross the whole retinal thickness and are radially connected (PR, photoreceptors; BP, bipolar cells; GC, ganglion cells). Other neural cells are amacrine cells (MA), horizontal cells (HC), and Müller cells (MC), which, in particular, terminate at the level of the outer limiting membrane (OLM). Vessels derived from the central retina artery form three different capillary beds were placed alongside the layers of nerve/ganglion layer, internal plexiform, and outer plexiform and interconnected by arterioles and capillaries. In **(B)**, the structure of the internal blood–retina barrier is given in detail, which consists of several histological elements: the adherent and tight junctions among the capillaries, the pericyte layers around endothelial cells, and extensions from the Müller cells, all of which take part in the control of metabolites and liquid exchange and endothelial proliferation. In **(C)**, the structure of the outer blood–retina barrier (oBRB) is represented and made from Bruch's membrane and the RPE layer, which play a pivotal role in controlling several parameters including internal pressure and ocular shape, oxygen exchange, and recycling of materials from the photoreceptor layer.

Among human tissues, the retina shows the highest oxygen consumption per unit weight to sustain elevated aerobic metabolism (10). In physiological conditions, the elevated blood flow is guaranteed by a dual arterial supply that allows independent vascularization of the outer and inner parts of the retina. Indeed, retinal layers are sandwiched between the outer and inner blood–retina barriers (oBRB and iBRB), which exhibit a very different anatomical structure (Figure 1). The retina from the external limiting membrane to the RPE is avascular and nourished by diffusion from the choroidal capillaries (11). Here, the oBRB is composed of the choroidal capillaries, the RPE, and Bruch's membrane, which are located between the basement membranes of the choroidal capillaries and RPE (6). The choroidal capillaries are fenestrated to provide a sustained intake of nutrients and adequate removal of waste products. In contrast, Bruch's membrane is formed by collagen and elastic sheets, and regulates the diffusion of nutrients based on their molecular weight. In addition, Bruch's membrane limits cell migration and controls intraocular pressure to randomize physical forces, thereby stabilizing the RPE layer. As a result, the RPE provides a wide exchange surface by means of an elevated number of microvilli, which extend between the outer parts of rods and cones, regulate nutrient supply, and recycle intracellular metabolites derived from the phagocytosed outer parts of photoreceptors. In addition, the RPE stabilizes the oBRB by providing vascular endothelial growth factor (VEGF) and other trophic factors to maintain the choroidal capillaries and their fenestrations. In a healthy individual, the most significant function of the choroidal circulation and the oBRB is to supply oxygen to photoreceptors, which are thought to consume more than 75% of retinal oxygen (12, 13). Notably, the pathological hallmark of DR is reduced oxygen exchange and consumption, followed by a low arteriovenous difference and abnormal venous oxygen saturation (14).

Blood, supplied from the central vessels of the retina which pass through the optic nerve, emerge at the papilla, and reach the internal surface of the retina. Unlike the outermost retinal layer, the innermost retinal layer is vascularized by three different plexuses organized alongside the layers of nerve/ganglion, internal plexiform, and outer plexiform. Similarly, the internal blood–retina barrier, which resembles the blood–brain barrier more, is consistently different from the oBRB (15). The capillaries do not show fenestrations but are continuous, and vicinal endothelial cells are joined together by adherent and tight junctions (Figure 1). In addition, endothelial cells are surrounded by pericytes even more covered by Müller cells, astrocytes, and microglial cells, which participate in the formation of the iBRB and collectively regulate endothelial cell proliferation and blood flow, providing additional trophic factors, antioxidants, and cytokines (16).

## Diabetic retinopathy

Diabetes mellitus is a leading pathology in the industrialized country, resulting in several serious life-threatening complications and death. It has been estimated that 1–5 Americans could be affected in 2050 (17). DM affects multiple organs like the retina, kidney, peripheral nerves, and blood vessels due to prolonged exposure to hyperglycemia caused by chronic and/or relative insulin insufficiency (18, 19). Vascular complications of diabetes are grouped into “macrovascular diseases,” associated with an increase in myocardial infarction and stroke, and “microvascular diseases” such as diabetic nephropathy, retinopathy, and neuropathy (20, 21). Of these, DR is the most prevalent illness among elderly people with diabetes living in developed countries. The prevalence of DR has reached 50% of patients with type 2 diabetes and 75% of patients with type 1 diabetes and remains a leading cause of moderate-to-severe vision loss and blindness worldwide (22). Other microvascular complications, such as diabetic nephropathy, have been shown to be major risk factors for macrovascular complications such as heart attacks and strokes (23–25).

In DR, prolonged hyperglycemia changes the structure of the retina and induces alterations to both neuronal and vascular cells, resulting in vision loss and blindness (26–28). In DR, new vessels grow in the normally avascular outer retina and in the subretinal space. Moreover, older patients with diabetes show impaired macular blood flow regulation that exacerbates diabetic retinal damage (29–31). In addition to vascular remodeling, inflammation, typically associated with type 2 diabetes, also seems to play a role in DR. This is suggested by the finding that only half of the patients were successfully treated with a specific anti-VEGF treatment (32). Accordingly, it is possible to detect an increase in several inflammatory markers in the blood serum, aqueous or vitreous humor, and ocular tissue of patients with DR. In particular, intercellular adhesion molecule 1 (ICAM-1), interleukin-1β (IL-1β), IL-6, IL-8, tumor necrosis factor α (TNF-α), and monocyte chemoattractant protein-1 (MCP-1) have been found to be increased in patients with DR (33, 34). Both neurons and glial cells are involved in the release of these inflammatory mediators, which can recruit leucocytes at the ocular level, further promoting the shift toward a pro-inflammatory environment. In addition, lymphokines and chemokines can also directly target endothelial cells, stimulating cell death and vasculature rearrangements.

The development of new diagnostic techniques has led to powerful improvements in the visualization of retinal structures and vasculature and, consequently, in the diagnosis of DR. Classically, the diagnosis of DR has been based on color fundus photographs and fluorescein angiography, injecting dye into a vein in the arm during a dilated eye examination. Nowadays, optical coherence tomography (OCT) imaging offers a rapid and non-invasive test by imaging cross-sectional pictures of the macula layers to detect retinal alteration that heralds the onset of DR (35).

Clinical examination of the retinal microvasculature defines the two major types of DR. Non-proliferative DR (NPDR) is characterized by the presence of microaneurysms, dot and blot hemorrhages, exudates, cotton wool spots, and intraretinal microvascular abnormalities, while proliferative DR (PDR) involves more extensive ischemia, neovascularization, and tractional retinal detachment, which is a high-risk factor for severe vision loss (36).

### Effect of diabetes and hyperglycemia on microvasculature and neurovascular units

The causative events underlying the pathogenesis of this disease are not completely understood. One of the most accredited hypotheses is that diabetes and hyperglycemia directly increase ROS production and alter the structure of the iBRB and oBRB events underlying vascular rearrangements (37, 38).

Briefly, the exposure of retinal cells to hyperglycemia triggers several related events, including massive glycation of cellular proteins, the formation of advanced glycation end products (AGEs), the formation of ROS, the release of pro-inflammatory cytokines followed by cell death—particularly in cells that are more exposed to hyperglycemia, such as pericytes (39). The loss of pericytes in the iBRB represents an early hallmark and is a breakthrough for DR because the disruption of the iBRB corresponds to deregulation in endothelial cell proliferation, leading to an outgrowth of dilated capillaries and microaneurysms, followed by vascular leakage and edema. Alternatively, it is frequently possible to find non-perfused or obliterated vessels with subsequent impaired flow and ischemia, followed by hypoxia-driven altered capillary regrowth (40).

Glycolysis is a metabolic pathway that produces adenosine triphosphate (ATP) under conditions that normally prevent ATP production by mitochondria. In healthy people with normal glycemia, glycolysis is fine-tuned to keep the intermediates stable. In diabetic patients with DR, hyperglycemia boosts glycolysis, leading to the accumulation of intermediates such as sorbitol *via* aldose reductase, as well as diacylglycerol (DAG) and AGEs (41). Increased DAG production during hyperglycemia activates protein kinase C-β (PKCβ), an isoform belonging to the PKC family active in vascular tissue (42), which consequently induces endothelial permeability, VEGF secretion, and inflammation (43). AGEs then bind to the ACE receptors, triggering VEGF expression and sustaining the pro-inflammatory event (44). In this view, VEGF has a central role during the inflammatory response in DR (45), as seen in other pathologies such as cardiovascular diseases like heart failure (46).

Being vascular remodeling one of the most dramatic events in DR, it is not surprising that in recent years, treatment of this pathology has been targeted at the retinal vessels: corticosteroids, laser photocoagulation, and anti-VEGF. Corticosteroids control inflammation and modulate genes encoding multiple inflammatory and anti-inflammatory proteins, leading to an amelioration of the BRB, even though it has also been demonstrated that corticosteroids can induce ocular hypertension and glaucoma (47). Because of the pivotal role of vascular outgrowth, laser photocoagulation has been a well-established treatment option for DR for more than half a century. Retinal oxygen demand is a regulator of angiogenesis, and this metabolic requirement can be reduced by laser photocoagulation, indicating that it could be an effective treatment for PDR (48). Potential side effects associated with this technique are moderate vision loss, diminished visual field, reduced color vision, and contrast sensitivity (49). In recent years, intravitreal injections of anti-VEGF drugs, such as ranibizumab, aflibercept, brolucizumab, aflibercept, or off-label drugs like bevacizumab, have become a common treatment for macular and retinal pathologies (50–52). However, these drugs have a short half-life and require regular intravitreal injections to maintain their efficacy, amplifying the risk of developing endophthalmitis caused by intravitreal injections of anti-VEGF agents (53, 54).

In addition to vascular remodeling, it is claimed that hyperglycemia can act directly and independently on neural cells. Using OTC analysis in addition to visual function tests [i.e., contrast sensitivity, perimetry testing, multifocal electroretinogram (mfERG), and dark adaptation], retinal thinning and visual dysfunction can be identified before the onset of DR, as has been demonstrated in diabetic patients without DR or with very early DR (55–57). This suggests a role for retinal neurodegeneration in the pathophysiology of DR. Retinal neurodegenerative events are common among species, as confirmed by animal models of diabetes. In mice and rats with autoimmune diabetes induced by the β-cell toxin streptozotocin, OTC analysis reveals thinning of the ganglion cell layer or inner plexiform layer, the inner/outer nuclear layer, as well as the entire retina (58, 59). Along with this neurodegeneration, vascular manifestation can be independent or at least concurrent. Indeed, in the ob/ob mouse model of type 2 diabetes, there was overall glial activation with leukostasis and a shift in microglia/macrophage phenotype before microvascular degeneration (60).

The breakdown of the inner BRB leads to loss of this complex neural environment and contributes to increased retinal vascular permeability and vision loss (61). Moreover, retinal microglia lose their motile cellular processes, became unresponsive to injuries, became denser, and had a smaller dendritic arbor (62). Retinal damage during DR further implicates retinal Müller glial cells and microglia as initiators of retinal inflammation. Purinergic signaling may explain this activation because of its well-established role in the immune-mediated inflammatory response in cardiovascular-related diseases (63). Purinergic signaling relies on the expression of receptors, i.e., purinergic P1 and P2 receptors, which recognize ATP, ADP, UTP, UDP, and nucleoside adenosine (ADO) molecules. Along with their function in the cell, these purine and pyrimidine molecules act as intercellular messengers. Indeed, after triggering of these receptors, subsequent cell signal transduction modulates tissue metabolism and normal physiology, but also the onset of pathological states of retinal diseases (64). Consistently, in DR, Müller cells amplify inflammation by releasing ATP in a CD40-dependent way, resulting in the activation of P2X7 purinergic receptors on retinal microglia, with subsequent expression of inflammatory cytokines, leading to neuroinflammation, vascular damage, and leakage (65).

## mTOR: An overview

**Figure 2:**
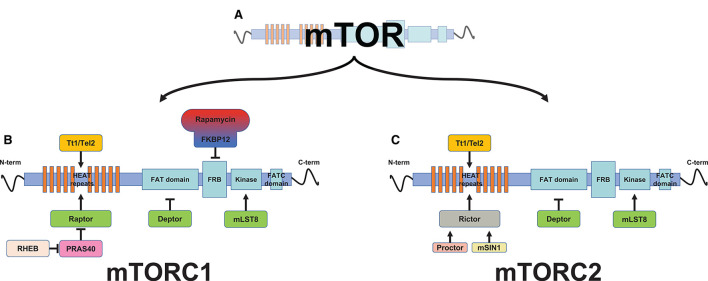
Schematic representation of mammalian target of rapamycin (mTOR) protein complexes. The mTOR protein **(A)** is associated with other proteins to form two distinct multiprotein complexes, mTOR complex 1 (mTORC1) **(B)** and mTORC2 **(C)**. mTOR is a multidomain protein kinase. From the N-terminus, the first domain of the mTOR protein is formed by a HEAT repeat (tandem-repeat protein domains) domain, which functions as protein-protein interaction surfaces for the substrate's recruitment proteins, Raptor (regulatory-associated protein of TOR, which define mTOR complex 1, mTORC1) and Rictor (rapamycin-insensitive companion of TOR, which define mTOR complex 2, mTORC2). Proctor (protein observed with Rictor) and mSin1 (mammalian stress activated protein kinase interacting protein 1) act as a Rictor activator. HEAT repeats further interaction with the Tt1/Tel2 complex (Tt1, Tel two interacting protein 1; Tel2, telomere maintenance 2, also described as HCLK2), thus stabilizing the mTOR protein. The Tt1/Tel2 complex (Tt1, Tel two interacting protein 1; Tel2, telomere maintenance 2, also described as HCLK2) interacts with the HEAT repeats, thus stabilizing mTOR. The FAT (FRAP, ATM, TRRAP) domain is the binding site for the mTOR inhibitor Deptor (the DEP domain containing a mTOR-interacting protein). The FRB (FKBP–rapamycin-binding) domain, which precedes the kinase domain, interacts with the inhibitory rapamycin *via* the immunophilin FKBP-12 (FK506-binding protein 12 kD). The mTOR kinase domain resides between the FRB and focal adhesion targeting C-terminal (FATC) domains, and shares a characteristic of both PI3K and canonical protein kinase families. Its kinase activity is enhanced by mLST8 (mammalian lethal with SEC13 protein 8). The latter domain of the mTOR protein is the FATC (FAT C-terminus) domain, located at the C-terminus of the protein.

The mechanistic (mammalian) target of rapamycin is a serine/threonine protein kinase involved in different diseases such as cancer, diabetes, and cardiac hypertrophy (66). The name derives from the identification of mTOR as the target of rapamycin, a macrolide antibiotic extracted from Streptomyces hygroscopicus in the 1970's. mTOR is a 289-kDa protein with multiple domains: HEATS repeats, the FAT domain, the FKBP12-rapamycin binding domain [FKBP–rapamycin-binding (FRB); the core domain that belongs to the phosphatidylinositol 3-kinase-related kinase family of protein kinase], and the focal adhesion targeting C-terminal (FATC) domain. The N-terminus HEATS is a docking site for the regulatory-associated protein (Raptor) and the rapamycin-insensitive companion of TOR (Rictor). The FAT domain binds to the regulatory protein Deptor; the FRB domain is the domain responsible for mTOR inhibition *via* the FKB-12-rapamycin complex; the C-terminus FATC domain is for substrate recognition and catalytic activity. mTOR interacts with several proteins to form two distinct signaling complexes, namely, mTORC1 and mTORC2, which are implicated in many cellular functions like cellular growth, metabolism, and autophagy in response to environmental cues (67). mTORC1 regulates metabolic pathways involving macromolecular synthesis, cell growth, and autophagy, while mTORC2 controls cell proliferation, survival, cytoskeletal remodeling, neovascularization, and autophagy (68, 69). mTORC1 is characterized by the association of mTOR with Raptor, together with other companion proteins, and by its sensitivity to rapamycin, while mTORC2 is insensitive to rapamycin (70), and Raptor has been replaced by Rictor, which is necessary for the mTORC2 substrate interaction (Figure 2).

Mammalian target of rapamycin complex 1 responds to growth factors, amino acids, and ATP levels (67). Growth factors, such as insulin, activate the phosphatidyl inositol 3-kinase (PI3K)/Akt cell signaling pathway. Akt phosphorylation by PDK1 carries out two events: the inactivation of the GTPase tuberous sclerosis protein (TCS)1/2 complex, which allows the Ras homolog enriched in the brain (Rheb) to accumulate in a GTP-bound form capable of binding to mTOR (71), and the disassociation of the inhibitor PRAS40 from Raptor, both of which events allow the activation of mTORC1 (72). Moreover, mTORC1 acts as a sensor of nutrients (amino acids) and energy (ATP) that enables protein synthesis, only when these components are available to support the metabolic requirement. Indeed, amino acids trigger the translocation of mTORC1 to the lysosomal surface where Rheb is located, activating mTORC1 *via* the Regulator complex (73). AMPK is a metabolic enzyme that acts as an indirect mTORC1 regulator, sensing the cellular AMP/ATP ratio. In the absence of an adequate amount of intracellular ATP, AMPK promotes the formation of the TSC1/2 complex, thereby increasing the inactive Rheb GDP-bound form and consequently inducing the inhibition of mTORC1 (74).

Mammalian target of rapamycin complex 2 responds to growth factors *via* phosphatidylinositol (3–5)-trisphosphate (PIP3), which is generated by PI3K in the cell membrane. The inhibition of mTORC2 is relieved upon the binding of PIP3 to the Pleckstrin homology domain of mSin1. By targeting Ser/Thr protein kinases (i.e., the AGC family of protein kinases and Akt), mTORC2 regulates cell migration through cytoskeletal remodeling and cell proliferation as well as apoptosis (67, 75, 76).

By virtue of their role as a sensor for the availability of nutrients and growth factors, mTORC1 and mTORC2 play physiological roles during embryonic development and tissue regeneration. Studies on animal models have shown that hematopoietic stem cells (HSCs) undergo long-term exhaustion as a result of mTOR ablation, enhancing their transition from G0 to G1 (77, 78). This exhaustion is higher in the high HSC ROS population where p38 inhibitor or rapamycin was able to restore HSC function (79).

Moreover, mTOR signaling plays a critical role in neuronal development, particularly in adult neurogenesis and neuronal atrophy (80). In the central nervous system, the mTOR pathway prevents apoptotic cell death, and this function is strictly linked with trophic factor activity (81). mTOR signaling is also involved in neurogenesis of the eye, and is highly activated in embryonic stages. Indeed, its temporal regulation is essential for the normal development of the retina and the optic nerve (82). Upon the deletion of TSC1, the activation of the mTOR pathway triggers the regeneration of adult retinal ganglion cells after optic nerve injury (83). This activity seems to be dependent on mTORC1 (84). Interestingly, in addition to its main role of regulating the production of red blood cells, erythropoietin (EPO) also has a role in neuroplasticity and neurogenesis after functional hypoxia (85), and this role seems to be linked to mTOR activity. Indeed, after oxygen-glucose deprivation, a condition that could cause some ischemia at the retinal level, EPO intimately regulates the mTOR pathway, preventing cellular injury and apoptotic events *via* the inhibition of PRAS40 (86). The relationship between EPO and mTOR could dependent on the Wnt pathway (87), as it has been documented that WISP1 activates mTORC1 through the phosphorylation of PRAS40 and TSC2 during microglial oxidative stress (88, 89). Moreover, the treatment of EPO also decreases mTOR expression and orchestrates the autophagy-related signaling pathways, suppressing cell injury in a rotenone-induced neurotoxicity model (90). In some cases, under hypoxic and superoxide stress, EPO promotes the survival of retinal progenitor cells by reducing autophagy (91) and becoming a promising neuroprotective agent for optic nerve protection and repair (92).

## mTOR and retinopathy

The retina is a high-demand site for oxygen and nutrients. During DR, in response to metabolic insults such as hypoxia, the retina undergoes morphological changes characterized by neovascularization with epiretinal vascular proliferation and subsequent vascular leakage and tractional retinal detachment. Early retinal pathophysiological modifications occur within the first few weeks of diabetes (93), and sustained hyperglycemia alters the distribution of oxygen around the retinal arterioles, inducing retinal vasculature rewiring, as is shown in a diabetes-induced rat model (94). Hypoxia modulates the angiogenic factor HIF-1α, an oxygen-sensitive transcription factor, to support retinal neovascularization. VEGF-α is a hypoxia-inducible gene target of HIF-1α. These proteins are downstream targets of mTORC1, as shown by rapamycin-directed suppression of hypoxia-inducible factors and vascular endothelial growth factors, followed by a reduction in vascularized tumor volume (95). In hyperglycemic rats for 8 weeks, an intraperitoneal injection of rapamycin reduces diabetes-induced VEGF overexpression that controls vascular permeability and angiogenesis (96). These observations are in line with the studies by Liu et al., in which the degree of retinopathy was mTORC1 dependent according to the expression of VEGF and PEDF proteins induced *via* the p-S6 protein in a DR rat model (97). Furthermore, VEGF may subsequently activate mTOR. Indeed, after the binding of VEGF to its receptor VEGFR-2, the PI3K/Akt pathway is activated and subsequently activates mTOR (98). This highlights that targeting the PI3K/Akt/mTOR signaling pathway could be a strategy to improve DR, as seen in human acute lymphoblastic leukemia (99).

Immunolocalization studies on human, rat, and mouse retinae have shown that the inner retina expresses mTORC pathways, with mTORC1 mainly localized to retinal ganglion cells and mTORC2 primarily relying on glial cells (100). As previously stated, the retina is a high-demand site for energy, and mTOR not only drives the perception of multiple upstream stimuli but also the regulation of cell metabolism and growth as downstream targets of PI3K. Indeed, the concurrent loss of mTORC1 and mTORC2 leads to inner and outer retinal morphology changes with a concurrent reduction in cone function, thus explaining the photoreceptor function loss observed during diabetes (101). In the retina, glial cells exert trophic support and influence programmed cell death, potentiating the neurodegeneration observed in retinal diseases (102). Experiments in an *in vitro* immortalized human Müller glial cell line and in an *in vivo* mouse-induced diabetic model show that the blockade of PPP1CA/YAP/GS/Gln/mTORC1 inhibits Müller cell proliferation and activation, suggesting a potential way to mitigate the development of DR (103).

### The crosstalk between mTOR and ROS in DR

Despite a wide body of literature focusing on ROS-induced microvasculature alterations, many studies report that retinal neurons can be directly targeted by diabetes, become an independent source of ROS, and undergo cell death, independently of and even before microvasculature alterations (104, 105).

It is well-known that hyperglycemia, as a consequence of nutrient overload, can promote oxidative stress through various metabolic pathways (5). Excessive amounts of ROS alter lipids, proteins, or deoxyribonucleic acid (DNA). NF-κB, acting as a redox sensor, plays a critical role in the regulation of the inflammatory response and programmed cell death (apoptosis) (5). In the retina, increased ROS production causes the activation of microglia with the expression of inflammatory cytokines, including IL-6, TNF-α, IL-1β, and IL-8, and adhesion molecules like ICAM-I and vascular cell adhesion molecule 1 (VCAM-1). Overall, these factors contribute to leukostasis and vascular leakage (106).

Yoshida et al. have shown that in a mouse model, hypoxia activates NF-κB in various retinal cell types (107). Furthermore, a recent *in vitro* experiment showed that NF-κB is activated in human retinal cells, including endothelial cells, pericytes, and astrocytes, under high glucose conditions (16). The overall effect of ROS can be ameliorated by oral administration of the natural phenol resveratrol, which reduces the level of inflammatory TNF-α and IL-6, in addition to activating NF-κB, in diabetic rat models (108, 109). This is in line with the effects observed by Bucolo et al. where the administration of antioxidant natural compounds, such as curcumin, carnosine, and α-lipoic acid, reduced the TNF-α and VEGF levels in the retinae of diabetic rats (110, 111). The mechanism of this inhibition could be explained by the downregulation of phosphorylation of NF-κB and the MAPK family in a mTOR-dependent manner, as shown in *in vitro* experiments conducted in lipopolysaccharide- (LPS-) stimulated microglial cells (112). Furthermore, it is well-known that ARPE-19, a human RPE cell line, under hyperglycemia presents metabolic changes including oxidative stress mediated by ROS (113). Yahg et al. have shown that the combined treatment of antidiabetic drugs, semaglutide, and rosiglitazone reduces high glucose-induced inflammatory injury by inhibiting ROS/PI3K/Akt/mTOR signaling pathway-related proteins TNF-α, IL-6, and IL-1β in ARPE-19, and enhances overall antioxidant capacity in a DR rat model by downregulating and upregulating, respectively, the levels of serum lipid peroxidation and superoxide dismutase (SOD) (114).

It is well-known that RPE cells play an essential role in maintaining the viability and functionality of photoreceptors, and that their loss of function results in alterations that are potentially causative of various retinal diseases (115). During pathogenic conditions, fully differentiated epithelial cells, *via* a process known as epithelial–mesenchymal transition (EMT), could reverse their phenotype to mesenchymal cells with invasive and migratory behavior toward the neuroretina, which in turn differentiate into fibroblasts/myofibroblasts. Next, the latter cell types could secrete excessive amounts of extracellular matrix components such as collagen (types I, III, IV, V, and VI) and fibronectin, resulting in fibrosis (116). Of note, during DR, EMT seems to be linked with the mTOR pathway and ROS, mainly driven by TGF-β. TGF-β has been found to be upregulated in the postmortem eyes of patients with ocular diseases and EMT, revealing a relevant role during the generation of DR (115, 117). Indeed, Kim et al. suggested a mechanism for mTOR activation and ROS generation with TGF-β, which contributes to EMT and fibrosis in retinal pigment epithelial cells (118).

Furthermore, recent findings in support of the involvement of the mTOR pathway showed that an mTOR inhibitor can modulate the expression of VEGF in the diabetic rat retina and VEGF-induced ROS enhancement in the Müller cell line (TR-MUL5) (96). These results are in line with the studies by Kim et al. where resveratrol limits the increase of VEGF, reducing early vascular lesions in diabetes-induced mouse retinae (119). L-glutamate is a major excitatory neurotransmitter in the nervous system, but excess extracellular glutamate may lead to neuronal and non-neuronal death and/or damage (120). In this context, ROS reduces glutamate clearance in the retina *via* the inhibition of glutamate intake in Müller cells, ultimately inducing retinal neurodegeneration (121). These effects are in line with the aforementioned relationship between ROS and mTOR, in the light of glutamate transporter 1 expression being a downstream target of mTOR (122).

From this point of view, it is clear that the observed relationship between oxidative stress and mTOR pathway in DR could be used to unveil potential new therapeutic opportunities to treat this illness.

### The crosstalk between mTOR and MicroRNAs in DR

MicroRNAs (miRNAs) are small, non-coding ribonucleic acids (RNAs) that regulate gene expression by pairing with complementary DNA sites and/or interfering with mRNA translation and stability (123). Although several studies have highlighted the crosstalk between the mTOR pathway and miRNA gene targeting (124, 125), few articles have investigated the role of miRNAs during DR. Among them, Li et al. have shown that the presence of ROS modulates the expression of miRNA-34a, increasing oxidative stress-related markers and cell apoptosis in ARPE-19 treated with high glucose (126). These findings are in line with a study by Liao et al. that miR-34a upregulates the phosphorylation of mTOR, which further reduces autophagy and enhances apoptosis in prostate cancer cells (127). However, further studies are needed to address the direct target(s) of miR-34 in regulating the mTOR pathway during DR.

Furthermore, in the treatment of ARPE-19 under high glucose conditions (50 mM), the overexpression of miR-130a exerts an antioxidant role by increasing the scavenger SOD1 levels in a TNF-α-dependent manner, as confirmed by the upregulation of TNF-α or knockdown of SOD1 (128). Although there is no clear evidence of the crosstalk between miR-130a and the mTOR pathway during DR, it is noteworthy that miR-130a is a negative regulator of TSC1, capable of upregulating the mTOR pathway in high-grade serous ovarian carcinoma (129). Then, the consequent aforementioned TNF-α upregulation and SOD1 phosphorylation could be the result of mTORC1 activation (114, 130). These findings could open up attractive new research areas for researchers involved in the study of DR. Currently, other miRNAs seem to be involved in the mTOR pathway during DR. During hypoxia, miR-7 is a critical mediator of the cellular response, reversing hypoxia-induced inhibition of mTOR signaling (131). Furthermore, it has been found that miR-7 can modulate cell proliferation by downregulating the expression of Hoxb3, mTOR, p-PI3K, and p-AKT in retinal epithelial cells (132).

Recent findings showed that the retinae of mice with DR notably decreased the expression of miRNA-29, and this event was associated with the inhibition of AMPK phosphorylation, with AMPK being the target protein of miR-29, and increased the expression of p-mTOR, thereby leading to excessive apoptosis observed during DR (133).

Lastly, the role of miRNA and mTOR pathways in DR needs further study, and bioinformatic analysis could be a useful tool to highlight their contribution to the pathophysiology of DR. Indeed, according to bioinformatic analysis, miRNA-204 with its three target genes Rictor, Dlg1, and SYNJ2BP, is associated with retinal diseases, suggesting that it has a relevant role in regulating Wnt signaling, the blood–retinal barrier, and angiogenesis (134).

### The crosstalk between mTOR and autophagy in DR

Autophagy is a catabolic process in which damaged cellular components are sequestered within a vacuole and degraded by fusing with lysosomes. Autophagy also allows cells to obtain free fatty and amino acids to sustain protein synthesis, and occurs as a selective process against specific organelles for disposal and tissue remodeling (135). Therefore, autophagy maintains adequate cellular homeostasis and energy levels. Autophagy can be distinguished into three main types: macroautophagy, microautophagy, and chaperone-mediated autophagy. Furthermore, autophagy discriminates targets in a specific and non-specific manner. Selective autophagy requires one or more receptors that tag targets for engulfment in the autophagosome, while non-selective autophagy is a bulk process that randomly picks up any kind of cytoplasmic proteins and ships it into the lysosome. In macro- and microautophagy, cytosolic components are engulfed in vacuoles and lysosomes through selective and non-selective mechanisms, while in chaperone-mediated autophagy, lysosome-associated membrane protein type 2A (LAMP-2A) first binds the substrate protein to the lysosomal membrane (136, 137).

Notably, the modulation of autophagy processes has been shown to represent an effective approach to the treatment of several human pathologies including neurodegenerative diseases (138, 139).

On the other hand, the modulation of autophagy is strictly dependent on the specific illness. Autophagy can play contrasting roles in different neurodegenerative diseases, playing an ameliorative role in some illnesses and contributing to the course of the disease in others.

In some neurodegenerative diseases, autophagy can act as a scavenger of misfolded and abnormally aggregated proteins, and in this context, autophagy stimulation can have a positive therapeutic role. Indeed, mTOR inhibition and autophagy activation have been shown to play a critical role in Alzheimer's disease. In Alzheimer's disease, mTOR activation promotes the production and accumulation of amyloid-β in the brain, and this event is linked with a direct inhibition of the autophagy-lysosomal system (140). Moreover, autophagy reduces the production of amyloid-β and ameliorates memory function in some animal models of Alzheimer's disease (141).

Similar findings are shown in neurogenerative Parkinson's disease where autophagic processes are dysfunctional with related accumulation of α-synuclein and other polyubiquitinated proteins (142).

Ischemia causes disorders related to nutritional needs and metabolic demands, and autophagy restores energy production *via* a catabolic process that allows neuronal cells to survive the nutrient depletion (143). In a neonatal model of hypoxia/ischemia, the inhibition of mTOR can activate autophagy (144). Moreover, in a model of spinal cord injury, rapamycin drives neuronal cell protection, promoting autophagy by inhibiting mTOR signaling (145).

Recently, Patergnani et al. reported alterations in glucose metabolism, impairment in mitochondrial functions, and excess of autophagy and mitophagy related to alterations in the mTOR/ULK1 pathway, in *in vitro, ex vivo*, and *in vivo* models of multiple sclerosis. The inhibition of autophagy with FDA-approved drugs strongly ameliorated axonal remyelination in all models and *in vivo* behavioral tests (146).

Similarly, in a mouse model of spinal cord injury, treatment with bisperoxovanadium was shown to activate the Akt/mTOR pathway, reduce autophagy, and rescue motor neurons from death (147).

Neurodegenerative disorders are significantly increasing worldwide. DR is now widely recognized as a neurodegenerative disorder (148), and its pathophysiology is closely related to the regulation of autophagy. Non-neuronal cells like RPE cells may also play a critical role in DR. RPE is part of the oBRB and regulates the transport of nutrients, water, and solutes from the choroid to the retina. In addition, RPE sustains photoreceptors and ensures the recycling of cones and rods that need to be replaced upon light absorption. Therefore, the autophagy of these cells appears to be relevant during DR. Consistently, Zhang et al. showed that high glucose conditions mediate the damage to ARPE-19 and increase its autophagy as well as apoptotic markers (p-p53, Bcl-2, and p62), and that these damages can be reversed by the autophagy inhibitor 3-methyladenine (3-MA), indicating a dysregulation of the autophagic process (149). This last observation was further confirmed by the same group, pointing out the beneficial effect of procyanidin, a member of the flavonoids, which inhibits autophagy (150). Interestingly, these beneficial effects were reversed when the autophagy agonist rapamycin was added to procyanidin treatment.

In DR, hypoxia and nutrient starvation increase circulating adipokines (i.e., leptin and adiponectin) to overcome metabolic deficiency. Recent findings indicate that adipokines may contribute to neovascularization during DR (151), a phenomenon linked with the mTOR pathway and the autophagy process. Li et al. found that in the rhesus choroid-retinal endothelial (RF-6A) cell model, during high glucose treatment, adiponectin promotes the expression of p-PI3K, p-AKT, and p-mTOR, increasing cell viability and lowering the autophagic process, therefore inhibiting high glucose-induced angiogenesis (152). Moreover, in the retina of DR mouse models, hypoxia and high glucose increase the expression of AGGF1 (an angiogenic protein with a function similar to VEGF-A) and promote autophagy with related angiogenesis. These phenomena were further confirmed *in vitro* using RF/6A cells, where the inhibition of the PI3K/AKT/mTOR pathway and the activation of autophagy-induced AGGF1-driven cell proliferation and tube-like structure formation (153).

As previously mentioned, hyperglycemia, acting as a major mechanism of DR pathology, causes neurodegeneration earlier than the detectable microvascular damage in which the mTOR/autophagy pathway is prominent. Recent studies on streptozotocin-induced diabetic models suggest that prolonged hyperglycemia downregulates mTOR-related proteins and GLUT1, with an increase of apoptotic markers as well as autophagic proteins in the ganglion cell layer. Blockage of autophagy by phlorizin (an insulin-independent glycemic control) and MHY1485 (an mTOR activator) normally rescues neuronal cells, suggesting that the mTOR pathway plays a relevant role associated with the damage to retinal ganglion cell (4).

Collectively, these observations highlight the dual nature of autophagy. In some circumstances, autophagy behaves as a protective mechanism, regulating inflammation, reducing starvation stress, and destroying noxious proteins. In others, it can worsen mitochondrial activity and energy replenishment, definitely targeting cells for death. This makes the dysregulation of autophagy a very interesting target in attempts to prevent the worsening of several illnesses, including DR.

Cellular responses like autophagy and senescence are closely related because many stresses including DNA damage, oxidative stress, and oncogenic stress can activate them. Both cellular responses prevent further proliferation of damaged cells, triggering cytotoxic or cytoprotective effects (154). Indeed, according to the level of autophagy, cells are driven to cell death or cellular senescence. It has been well-described that the exposure of ARPE-19 cells to a high concentration of glucose alters metabolism and increases overall ROS production and lipid accumulation, contributing to senescence (155, 156). Thus, Chae et al. reported interesting results: in a doxorubicin-induced mouse model of RPE senescence, they found that selective targeting of senescent RPE cells by Nutlin-3a ameliorates age-related macular degeneration (157). This finding may appear tricky, but it is noteworthy that although p53 is an autophagy agonist *via* the inhibition of mTORC1, Nutlin-3a causes quiescence and senescence program suppression (158, 159). Overall, in RPE, given that mTOR and p53 are key mediators of autophagy and senescence responses, this may represent an attractive target to eliminate senescent cells in DR.

Along with non-retinal cells that are affected by DR, Müller cells and retinal microvascular endothelial cells have been implicated in altering autophagic processes. Müller cells respond to vascular injury, trauma, and metabolic stresses by releasing trophic factors (i.e., VEGF) and phagocytosing degenerated cells to maintain retinal homeostasis (160). *In vitro* experiments have shown that upon high glucose stress, Müller cells increase autophagic markers with the accumulation of p62/SQTSM1. Despite this process, glial cells undergo programmed cell death and release massive amounts of VEGF. On the other hand, rapamycin restores the autophagic machinery and protects cells from apoptosis, thus highlighting the role of autophagic dysfunction in these cells during DR (161). Along with these alterations, Müller cells undergo the dysregulation of mitophagy and become more susceptible to redox stress (162).

Therefore, the induction of autophagy seems to play a relevant role in maintaining cell survival in the nervous system, and mTOR is a conductor for autophagic activity in the cells, making it a candidate for the crosstalk between the mTOR pathway and autophagy as an attractive option to manage DR.

## Conclusion

Diabetic retinopathy is a complex disease without a completely clarified etiology. Diabetes at the intracellular level prompts oxidative stress and redox equilibrium imbalance through different cellular and mitochondrial pathways. The successive cellular alterations and death lead to profound changes in the histology of the retina, with malfunction and loss of photoreceptors and other neural cells. In parallel, weakening the BRB leads to microvascular changes that reduce the availability of oxygen to photoreceptors. This impacts the survival of neurons and, in the meantime, induces a marked trophic factor-dependent redrawing of the retinal microvascular structure, as is typical of DR.

Mammalian target of rapamycin coordinates multiple anabolic and catabolic processes involved in promoting cell growth and acts as a sensor for growth factors and nutrients. The finding reported in this review highlights that in neurodegenerative diseases like retinopathy, the mTOR pathway can be over activated or inhibited. Furthermore, corticosteroids, laser photocoagulation, and anti-VEGF therapy are becoming the standard of care for the treatment of DR, but can have adverse problems or encounter non-responding subjects, preventing their use in some patients.

**Figure 3:**
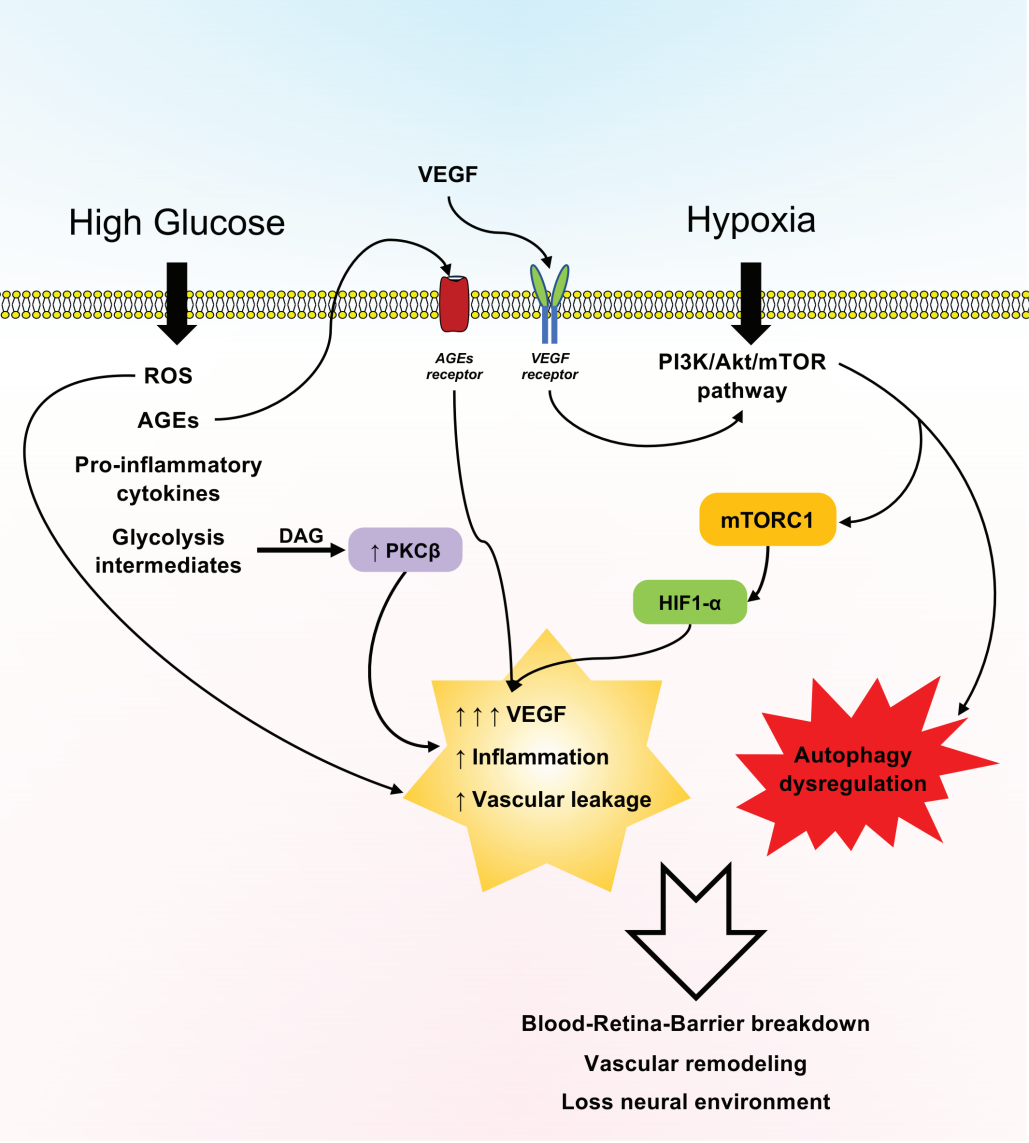
Schematic representation of the effects of the mTOR pathway in diabetic retinopathy (DR). Hyperglycemia and hypoxia prompt a plethora of effects associated with the expression of the PI3K/Akt/mTOR pathway, reactive oxygen species (ROS), advanced glycation end products (AGEs), inflammatory cytokines, and glycolysis intermediates, which in turn sustain vascular leakage and overall inflammation of the retina, with the loss of the blood–retina barrier and the neural microenvironment being mainly driven by increased vascular endothelial growth factor (VEGF) production and dysregulated autophagy.

Concurrently, this review highlights that in DR, the mTOR pathway seems to be involved in a plethora of effects linked to oxidative stress, autophagy dysregulation, and cell death, as seen in various experimental models (Figure 3). In this opinion, although knowledge gaps deserve further elucidation, mTOR targeting in particular could be an attractive target for researchers to postulate novel therapies to treat DR.

## Author contributions

GZ and FC: conceptualization. FC, EZ, and MP: writing—original draft preparation. FC, ER, and MP: writing—review and editing. FC: figures preparation. FC, ER, MP, MM, MB, and GZ: revision process and final editing. All authors have read and agreed to the published version of the manuscript.

## Funding

This manuscript was supported by local funds from the University of Ferrara grant numbers: 2020-FAR.L-CF_003 and 2021-FAR.L-CF_002.

## Conflict of interest

The authors declare that the research was conducted in the absence of any commercial or financial relationships that could be construed as a potential conflict of interest.

## Publisher's note

All claims expressed in this article are solely those of the authors and do not necessarily represent those of their affiliated organizations, or those of the publisher, the editors and the reviewers. Any product that may be evaluated in this article, or claim that may be made by its manufacturer, is not guaranteed or endorsed by the publisher.
